# Multiply Broken Metallic Hardware Removal for United Patella Fracture Applied Twenty Years Before: A Case Report

**DOI:** 10.31729/jnma.5625

**Published:** 2020-11-30

**Authors:** Kapil Mani KC, Parimal Acharya, Bandhu Ram Pangeni, Ankit Niroula, Amuda KC

**Affiliations:** 1 Civil Service Hospital, Minbhawan, Kathmandu, Nepal; 2 Nepalese Army Institute of Health Sciences, Sanobharyang, Kathmandu, Nepal

**Keywords:** *broken wires*, *cerclage wires*, *migration*, *patella fracture*, *tension band wiring*

## Abstract

Breakage of tension band wires, used to treat the patella fracture, is not uncommon several years after the fracture fixation. Broken wires may migrate to surrounding neurovascular structures, other vital organs like heart and may cause potentially fatal complications. Once the wires have been broken, it is very difficult to remove the broken pieces of metal wires. We report a 50 years old male patient with broken tension band wires at multiple sites for patella fracture. The broken wires were removed 20 years after the initial surgery without any undue complications, however patient sustained significant soft tissue damage to remove all the pieces of broken wires that would otherwise have been removed without any undue complications immediately after fracture union.

## INTRODUCTION

Tension band wiring (TBW) with stainless steel wire is a gold standard technique to treat the patella fractures.^1^ However breakage of wires is not uncommon several years after fracture fixation. Broken wires may migrate towards the surrounding soft tissue, neurovascular structures, other major organs like heart and causes potentially fatal complications.^2,3,4^ So it seems worthful to remove the wires after fracture has been united. Once wire has been broken it is really tough to remove the implants and causes significant amount of soft tissue damage that would otherwise has been removed easily. On this back ground we would like to address ideal time to remove the tension band wires in patella fracture.

## CASE REPORT

A 50 years old male patient came to our hospital with complain of prominence of hardwares and occasional pain on left knee. He sustained patella fracture 20 years before and was performed tension band wiring in some other hospital at that time. The fracture was united without any undue complications and patient had no problems for past so many years. Even though the operating surgeon advised him to remove the implant after certain time period, he did not follow the suggestions and simply engaged with his own activities.

**Figure 1:**
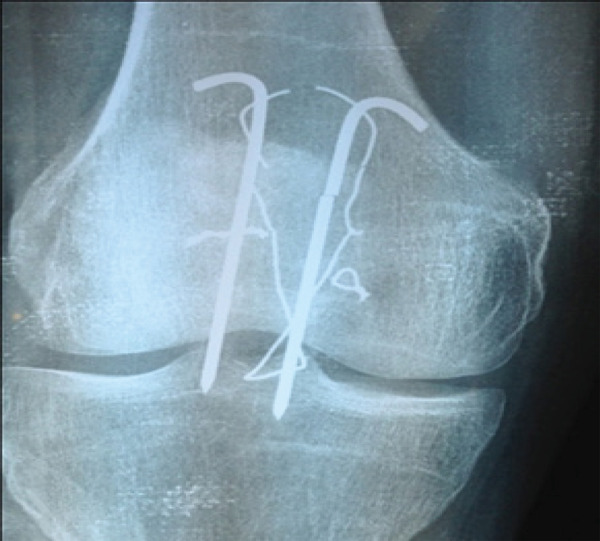
Antero-posterior view of knee joint showing multiple pieces of broken stainless steel wires and K wires.

However he was feeling some discomfort and prominence of implants for last five years. On examination there was prominent hardwares and diffuse tenderness on anterior surface of knee but there was no swelling, bursa formation and deformity of knee joint. X-ray of knee joint antero-posterior and lateral views showed that there were multiple broken pieces of tension band wires on anterior aspect of patella (Figure 1).

**Figure 2:**
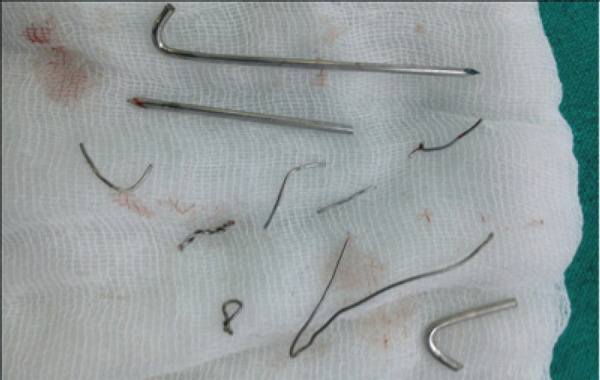
Multiple broken pieces of Kirschner wires and stainless steel wires after removal from the patella.

Patient was posted to operation theatre for removal of implant. Incision was given in the previous transverse scar site in the knee joint. All the broken pieces of wires were removed one by one with the assistance of C arm. However surgery was very tedious, multiple incisions were given on pre-patellar soft tissue that caused significant amount of soft tissue compromise. It almost took one and half hour to remove all the pieces of wires (Figure 2). Patient was allowed partial weight bearing with the assistance of crutches second day after surgery. Stiches were removed after 2 weeks and patient was walking normally without pain two months after removal of implant.

## DISCUSSION

The behaviour of retained implants in patella fracture is different as compared to those in other bone fractures. In case of long bones once the fracture has been united, the implants do not share the loading forces and they are not prone to be broken even after prolong time interval. However tension band and cerclage wires inside the quadriceps and patellar tendon in case of fractured patella are subjected to repetitive strain and loading forces even after the fracture has been healed. So, on due course of time these metal wires may eventually break if not removed.^1^ Some authors still believe that asymptomatic implants can be retained indefinitely even in the united patella fractures.^5^

Once the fracture was united, patients sometimes may not be interested to remove the implants for long period of time unless they are troubled by pain and some other serious complications. In such cases tension band and cerclage wires are prone to break and broken metal wires not only cause impingement on the skin and formation of bursa but also migrate to surrounding soft tissues due to repeated movement of knee joint. There are many reports in the literature regarding the migration of K wires to heart from proximal humerus, distal radius, hip joint, sternoclavicular joint and acromioclavicular joint.^6^ Biddau et al reported the migration of broken cerclage wires from patella into the heart 13 years after the initial surgery.^7^ Similarly Choi et al reported the migration of broken wires from fixed patellar fracture to the popliteal fossa and Hsu et al postulated intra-articular migration of broken wires from the patella.^3,4^ The study of Mak Nin and Tai Sammy showed that there was no statistically significant difference between the age of patient in broken wire groups and intact wire groups.^1^ However difference was statistically significant for length of time from fracture fixation to removal of implant between two groups of patients. Younger populations had higher cumulative risk of wire breakage as compared to the elderly population probably due to their longer life span rather than the activity level.^1^ Their study further quoted that incidence of wire breakage increases to the patients whose wires were removed more than or equal to 12 months. Repetitive loading forces and strain of both quadriceps and patellar tendon, even after the fracture united predispose to wire breakage. However use of relatively large diameter wire, application of wires applying the AO principles and use of good quality wire with higher modulus of elasticity prevents the wire breakage to some extent.^8^

In our case we removed the multiple pieces of broken wires in united fracture patella 20 years after the initial surgery. It is one of the rare cases reported in the literature whose broken wires were removed in large time interval. Fortunately broken wire pieces did not migrate to surrounding neurovascular structures and other vital organs that would otherwise cause fatal complications. However we had to give multiple incisions in the soft tissue to remove all the pieces of wires that compromised the post-operative rehabilitation of patients even more than that during application of wires at the time of patella fracture.

So we strongly recommended to remove the metal wires after union of patella fracture not more than or equal to12 months after initial surgery.
